# Multisynaptic Projections from the Amygdala to the Ventral Premotor Cortex in Macaque Monkeys: Anatomical Substrate for Feeding Behavior

**DOI:** 10.3389/fnana.2018.00003

**Published:** 2018-01-19

**Authors:** Hiroaki Ishida, Ken-ichi Inoue, Masahiko Takada

**Affiliations:** ^1^Frontal Lobe Function Project, Tokyo Metropolitan Institute of Medical Science, Tokyo, Japan; ^2^Systems Neuroscience Section, Primate Research Institute, Kyoto University, Inuyama, Japan

**Keywords:** amygdala, feeding behavior, primates, rabies virus, ventral premotor cortex

## Abstract

The amygdala codes the visual-gustatory/somatosensory valence for feeding behavior. On the other hand, the ventral premotor cortex (PMv) plays a central role in reaching and grasping movements prerequisite for feeding behavior. This implies that object valence signals derived from the amygdala may be crucial for feeding-related motor actions exerted by PMv. However, since no direct connectivity between the amygdala and PMv has been reported, the structural basis of their functional interactions still remains elusive. In the present study, we employed retrograde transneuronal labeling with rabies virus to identify the amygdalar origin and possible route of multisynaptic projections to PMv in macaque monkeys. Histological analysis of the distribution pattern of labeled neurons has found that PMv receives disynaptic input primarily from the basal nucleus, especially from its intermediate subdivision. It has also been revealed that the medial (e.g., the cingulate motor areas, CMA) and lateral (e.g., the insular cortices) cortical areas, and the cholinergic cell group 4 in the basal forebrain probably mediate the projections from the amygdala to PMv. Such multisynaptic pathways might represent amygdalar influences on PMv functions for feeding behavior.

## Introduction

The amygdala is composed of a structurally heterogeneous collection of subnuclei, including the basal (B), accessory basal (AB), lateral (L), central (C) nuclei (Amaral et al., 1992). There is a consensus that multimodal projections from the visual, auditory, somatosensory and visceral cortices are directed primarily toward L and B (Webster et al., 1991; Stefanacci and Amaral, 2000, 2002; Yukie, 2002; Amaral et al., 2003), and that B as well as AB mainly receives such inputs from L and, in turn, sends output projections to C (Pitkänen and Amaral, 1998). Further, it is generally accepted that B and AB give rise to widespread cortical projections, whereas C constitutes a principal origin of subcortical projections (Amaral and Price, 1984; Carmichael and Price, 1995; McDonald, 1998; Stefanacci and Amaral, 2002). Among the cortical areas that communicate with B and AB, the posterior orbitofrontal cortex and the anterior cingulate cortex are the two major areas, both of which have been implicated in reward values (Thorpe et al., 1983; Rolls and Baylis, 1994; Devinsky et al., 1995; Shima and Tanji, 1998b; Rolls, 2000, 2005; Matsumoto et al., 2003; Buckley et al., 2009). The motor-related areas of the frontal lobe, such as the cingulate motor areas (CMA), supplementary and presupplementary motor areas (SMA, pre-SMA), and premotor cortex (especially its rostrodorsal part), also receive direct projections from B and AB, though weaker than the projections from the posterior orbitofrontal and anterior cingulate areas (Pandya et al., 1973; Jacobson and Trojanowski, 1975; Porrino et al., 1981; Avendaño et al., 1983; Amaral and Price, 1984; Barbas and De Olmos, 1990). Seminal single-unit recording studies have demonstrated that these motor-related areas are involved in goal-directed actions based on reward (Niki and Watanabe, 1979; Shima and Tanji, 1998b; Pastor-Bernier and Cisek, 2011). Several lines of evidence suggest that the amygdala-derived pathways to the motor-related areas may represent valence signals of sensory stimuli for driving goal-directed behavior in emotional and motivational contexts (Salzman and Fusi, 2010; Barbas et al., 2011; Grèzes et al., 2014). In fact, many pioneer works have shown that neurons in the amygdala code the visual-gustatory/somatosensory valence for feeding behavior (Sanghera et al., 1979; Fukuda et al., 1987; Nishijo et al., 1988a,b). These electrophysiological findings indicate that the amygdala play a key role in evaluating the valence of sensory stimuli (see also Weiskrantz, 1956; Jones and Mishkin, 1972; Gaffan and Harrison, 1987; Gaffan et al., 1988).

The ventral premotor cortex (PMv) has repeatedly been shown to play a central role in reaching/grasping movements prerequisite for feeding behavior (Rizzolatti et al., 1981, 1988; Halsband and Passingham, 1985; Murata et al., 1997; Graziano et al., 2002; Kurata and Hoshi, 2002). It has also been reported that neurons in the monkey PMv encode motivational signals for rewarded actions (Roesch and Olson, 2003, 2004). Thus, object valence signals derived from the amygdala would be indispensable for feeding-related motor actions exerted by PMv. However, the structural basis of functional interactions between the amygdala and PMv still remains elusive, because PMv receives no direct input from the amygdala. In the present study, we therefore employed retrograde transneuronal labeling with rabies virus to identify possible multisynaptic projections from the amygdala to PMv in macaque monkeys. By injecting the virus into the forelimb region of PMv, we analyzed the distribution pattern of retrogradely labeled neurons within the amygdala and explored the entire architecture of their linkage to PMv.

## Materials and Methods

We used four male macaque monkeys (Macaca fuscata, weighing 5.4–6.9 kg; provided by the Primate Research Institute, Kyoto University, Table 1) who were the same subjects as used in our previous report (Ishida et al., 2016). The experimental protocol was approved by the Animal Welfare and Animal Care Committee of the Primate Research Institute, Kyoto University, and all experiments were conducted in accordance with the Guideline for the Care and Use of Animals of the Primate Research Institute, Kyoto University.

**Table 1:** Summary of experiments.

Monkey	Species	Injection site	Tracer	Survival (days)	Injection tracks (*n*)	Injection volume (μl)
Case 1	*M. fuscata*	PMv	CVS-11	3 (71 h)	2	2.0
Case 2	*M. fuscata*	PMv	CVS-11	3 (72 h)	2	2.0
Case 3	*M. fuscata*	PMv	CVS-11	4 (92.5 h)	2	2.0
Case 4	*M. fuscata*	PMv	CSV-11	4 (96 h)	2	2.0

### Surgical Procedures

Monkeys were subjected to general anesthesia induced with ketamine hydrochloride (10 mg/kg, i.m.) and maintained with sodium pentobarbital (20 mg/kg, i.v.). During the surgical operation, monkeys were kept hydrated with lactated Ringer’s solution (i.v.). An antibiotic (Rocephin; 75 mg/kg, i.m.) and an analgesic (Buprenex; 0.01 mg/kg, i.m.) were administered at the time of initial anesthesia. Each monkey’s head was secured in a stereotaxic frame, and the skin and muscle were retracted to expose the skull over the right hemisphere. A craniotomy was made over the right frontal cortex, and the dura mater was cut to expose the superior and inferior limbs and the genu of the arcuate sulcus, which allowed us to visually inspect the tracer injection sites at the cortical surface. After confirming this, we proceeded with tracer injections.

### Viral Injections

**Figure 1:**
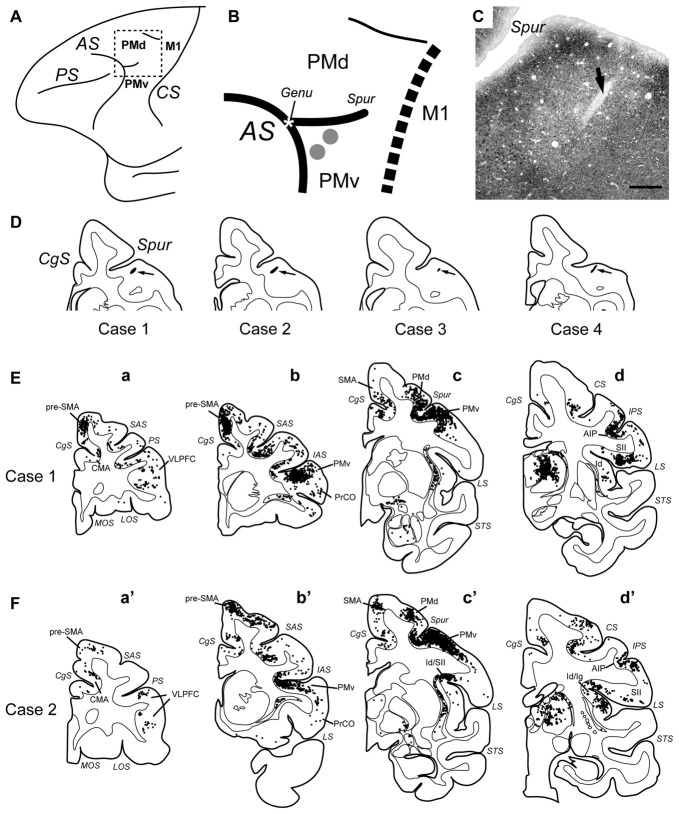
**(A)** Schematic diagram showing the frontal lobe of the macaque monkey. The rectangular area demarcated with broken lines is enlarged in **(B)**. AS, arcuate sulcus; CS, central sulcus; M1, primary motor cortex; PMd, dorsal premotor cortex; PMv, ventral premotor cortex; PS, principal sulcus. **(B)** Sites of rabies injections in PMv. The estimated viral spread around each injection needle track is indicated with the gray circle (1 mm in diameter). The genu of AS (Genu) is denoted with the asterisk. The border between the premotor areas and M1 is drawn with the dotted line. Spur, spur of AS. **(C)** Photomicrograph of the injection site in Case 4. The arrow denotes the injection needle track. Scale bar, 1 mm. **(D)** Drawing of a representative coronal section through the injection needle track (pointed to by the arrow) in each case. CgS, cingulate sulcus. **(E,F)** Distributions of retrograde neuron labeling 3 days after the rabies injections into PMv in Case 1 **(E)** and Case 2 **(F)**. Only cortical labeling is depicted. In each case, four representative coronal sections are arranged rostrocaudally in **(a–d)** or **(a′–d′)**. Each dot represents one labeled neuron. AIP, anterior intraparietal area; CMA, cingulate motor areas; Id, dysgranular insular cortex; Ig, granular Insular cortex; IAS, inferior limb of AS; IPS, intraparietal sulcus; LOS, lateral orbital sulcus; LS, lateral sulcus; MOS, medial orbital sulcus; PrCO, precentral operculum region; pre-SMA, presupplementary motor area; SAS, superior limb of AS; SMA, supplementary motor area; STS, superior temporal sulcus; SII, secondary somatosensory cortex; VLPFC, ventrolorateral prefrontal cortex.

Rabies virus (CVS-11 strain; 1.0 × 108 focus-forming units/ml) was derived from the Centers for Disease Control and Prevention (Atlanta, GA, USA) and donated by Dr. S. Inoue (The National Institute of Infectious Diseases, Tokyo, Japan). Concerning viral injections and injection sites, two tracks of injections of rabies virus were made into the forelimb region of PMv for each of the four monkeys (Figures 1A–D; Table 1). A viral suspension was slowly injected through a 10-μl Hamilton microsyringe. Along each injection track, viral deposits were placed at two different depths: 3 and 2 mm below the cortical surface. At each depth, 0.5 μl of the viral suspension was deposited. When injections were complete, the dura mater and bone flap were repositioned, and the scalp incision was closed.

### Histology

With survival periods of 3 days (71 h, case 1; 72 h, case 2; for the second-order labeling) or 4 days (92.5 h, case 3; 96 h, case 4; for the third-order labeling) after viral injection, monkeys were deeply anesthetized with an overdose of sodium pentobarbital (50 mg/kg, i.v.) and transcardially perfused with 10% formalin in 0.1 M phosphate buffer (pH 7.4). The fixed brains were removed from the skull, postfixed in the same fresh fixative overnight at 4°C, and placed in 0.1 M phosphate buffer (pH 7.4) containing 30% sucrose. Coronal sections were then cut serially at 50 μm thickness on a freezing microtome. Every sixth section was processed for immunohistochemical staining for rabies virus by means of the standard avidin-biotin-peroxidase complex method. Following immersion in 1% skimmed milk, the sections were incubated overnight with rabbit anti-rabies virus antibody (donated by Dr. S. Inoue) in 0.1 M phosphate-buffered saline (pH 7.4) containing 0.1% Triton X-100 and 1% normal goat serum. The sections were then placed in the same fresh incubation medium containing biotinylated goat anti-rabbit IgG antibody (diluted at 1:200; Vector Laboratories, Burlingame, CA, USA), followed by the avidin-biotin-peroxidase complex kit (ABC Elite; Vector Laboratories). To visualize the antigen, the sections were reacted in 0.05 M Tris-HCl buffer (pH 7.6) containing 0.04% diaminobenzidine, 0.04% nickel chloride and 0.002% hydrogen peroxide. The sections were mounted onto gelatin-coated glass slides and then examined under a light microscope (Nikon Eclipse 80i, Tokyo, Japan).

### Data Analysis

We digitized the outline of the nuclei of the amygdala and the location of labeled neurons with the MD-Plot 5 system (Accustage, Shoreview, MN, USA) attached to the microscope system. Neuronal labeling was plotted on tracings of equidistant coronal sections (each 300 μm apart) throughout the amygdala. The distribution of labeled neurons in specific nuclei of the amygdala was determined by superimposing plots of neuronal labeling on adjacent Nissl-stained sections. According to the criteria described by Amaral and Price (1984) and Amaral et al. (1992), the amygdala was classified into the basal (B), AB, lateral (L), central (C) and other nuclei. The B was further subdivided into magnocellular (Bmc), intermediate (Bi) and parvocellular (Bpc) parts.

### Safety Issues

Experiments involving rabies virus were performed in a special primate laboratory (biosafety level 2) designated for *in vivo* infectious experiments. Throughout the experiments, the monkeys were housed in individual cages that were installed inside a special biosafety cabinet. To avoid accidental infection with the virus, all investigators received immunizations beforehand and wore protective clothing during the experimental sessions. Equipment was disinfected with 80% (v/v) ethanol after each experimental session, and waste was autoclaved prior to disposal.

## Results

### Rabies Injections into PMv

The injection sites were anatomically determined based on previous electrophysiological results showing that a sector of PMv located just ventral to the genu of the arcuate sulcus plays a crucial role in reaching movement (Hoshi and Tanji, 2002, 2006). Since this portion of PMv has been shown to receive no direct projections from the amygdala (Jacobson and Trojanowski, 1975; Avendaño et al., 1983; Amaral and Price, 1984), we employed retrograde transneuronal labeling with rabies virus. In each animal, two injection tracks (approximately 1 mm apart) were targeted at this portion of PMv; the injection sites were situated 1–2 mm posterior to the genu of the arcuate sulcus and 1–2 mm lateral to the spur of the arcuate sulcus (Figures 1A–D).

Three days after the rabies injections into PMv, the labeled neurons were seen in the internal segment of the globus pallidus (GPi), but not in the external segment of the globus pallidus (GPe) within the basal ganglia (see also Ishida et al., 2016). This indicates that the 3-day postinjection period resulted in the second-order, as well as the first-order, neuron labeling across two synapses. Four days after the rabies injections into PMv, labeled neurons were further found in GPe and the striatum. This implies that the 4-day postinjection period yielded the third-order neuron labeling across three synapses.

At the 3-day postinjection period, retrogradely labeled neurons including mono/disynaptic neurons were seen in a variety of cortical areas. First, the labeled neurons were densely observed around the injection sites including the rostral and lateral aspects of PMv (corresponding to sections **b** and **b′** in Figures 1E,F). A number of labeled neurons were also located in the caudal aspect of the dorsal premotor cortex (corresponding to sections **c** and **c′** in Figures 1E,F). Second, the labeled neurons were found in the medial cortical areas (e.g., the CMA, SMA and pre-SMA); corresponding to sections **a–c** and **a′–c′** in Figures 1E,F), and in the lateral cortical areas (e.g., the ventrolorateral prefrontal cortex (VLPFC), precentral operculum region (PrCO), granular and dysgranular insular cortices (Ig/Id), anterior intraparietal area (AIP), and secondary somatosensory cortex (SII); corresponding to sections **a–d** and **a′–d′** in Figures 1E,F). Previous anatomical studies have shown that these cortical areas have direct connections with PMv (Matelli et al., 1986, 1998; Kurata, 1991; Morecraft and Van Hoesen, 1992; Luppino et al., 1993, 2003; Simonyan and Jürgens, 2002, 2005; Dum and Strick, 2005; Gerbella et al., 2011).

**Figure 2:**
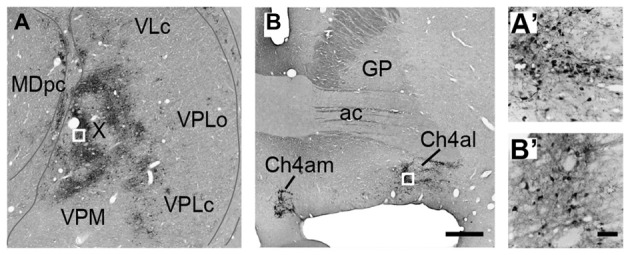
Photomicrographs of coronal sections through the thalamus **(A)** and the basal forebrain **(B)** in Case 1. Retrograde neuron labeling 3 days after the rabies injections into PMv. Boxed areas in **(A,B)** are enlarged in **(A′,B′)**, respectively. Scale bars in **(B,B′)**, 1 mm for **(A,B)**, and 100 μm for **(A′,B′)**. ac, anterior commissure; Ch4al, antero-lateral territory of the cholinergic cell group 4; Ch4am, antero-medial territory of the cholinergic cell group 4; GP, globus pallidus; MDpc, parvocellular division of the mediodorsal thalamic nucleus; VLc, caudal division of the ventrolateral thalamic nucleus; VPLc, caudal division of the ventroposterolateral thalamic nucleus; VPLo, oral division of the ventroposterolateral thalamic nucleus; VPM, ventroposteromedial thalamic nucleus; X, area X.

In the thalamus, neuronal labeling was found in the ventral nuclei, area X (Olszewski, 1952; Paxinos et al., 2000), and the parvocellular division of the mediodorsal nucleus (MDpc; Figures 2A,A′). These areas have been reported to possess direct connections with PMv (Matelli et al., 1986; Holsapple et al., 1991; Morel et al., 2005). At the same survival period, the labeled neurons were seen in the basal forebrain in which the cholinergic cell group 4 (Ch4), mainly its antero-lateral/medial territory (Ch4al/am) and additionally its intermedio-dorsal/ventral territory (Ch4id/iv), contained labeled neurons Figures 2B,B′).

**Figure 3:**
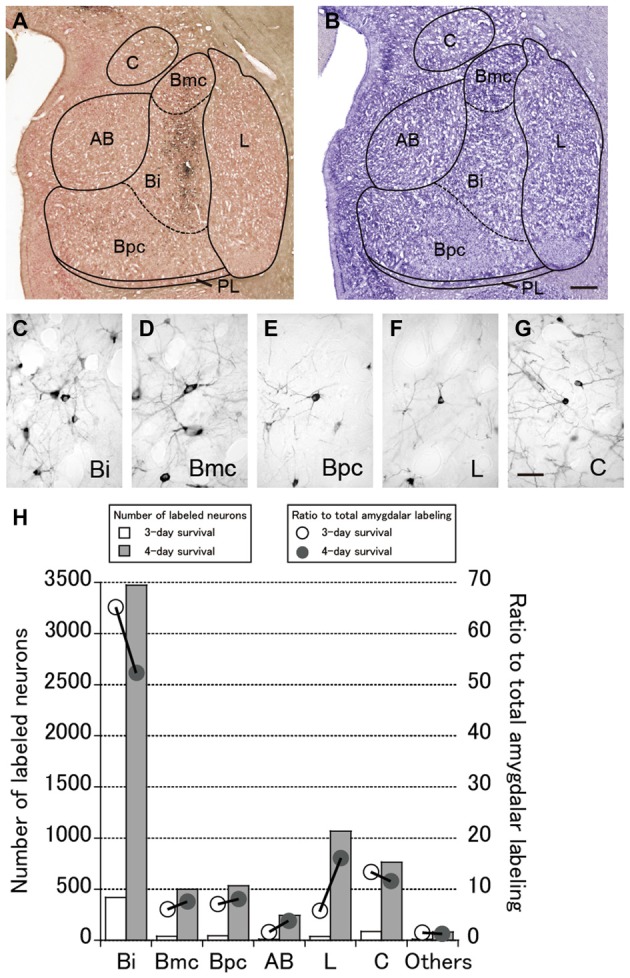
**(A)** Low-magnification photomicrograph of a representative coronal section through the amygdala in Case 3 in which the monkey was sacrificed 4 days after the rabies injections into PMv. **(B)** Nissl-stained section adjacent to the section in **(A)**. AB, accessory basal nucleus; Bi, intermediate division of the basal nucleus; Bmc, magnocellular division of the basal nucleus; Bpc, parvocellular division of the basal nucleus; C, central nucleus; L, lateral nucleus; PL; paralaminar nucleus. Scale bar in **(B)**, 1 mm for both panels. **(C–G)** Examples of labeled neurons in the amygdalar subnuclei. Scale bar in **(G)**, 50 μm for all panels. **(H)** Summary histograms showing the number (squares) and ratio (circles) of labeled neurons at the 3-day (open symbols) and 4-day (filled symbols) survival periods. The mean values in each subnucleus are indicated.

Numbers of neurons were labeled in the amygdala. These neurons were considered to project to PMv across synapses because of the lack of a direct projection from the amygdala to PMv. Figure 3A represents an example of the overall distribution of labeled neurons in the amygdala at the 4-day postinjection period. Shown in Figure 3B is a section adjacent to that in Figure 3A on which the subnuclei of the amygdala are identified with their borders (Amaral and Price, 1984; Amaral et al., 1992). Figures 3C–G depict examples of labeled neurons in several representative subnuclei. The number of labeled neurons in each subnucleus and their ratio to the total amygdalar labeling are summarized in Figure 3H. Below we describe the distribution patterns of labeled neurons within the amygdala in the monkeys who were allowed to survive for 3 or 4 days after the rabies injections.

### Labeling of Amygdalar Neurons 3 Days after Rabies Injections into PMv

**Figure 4:**
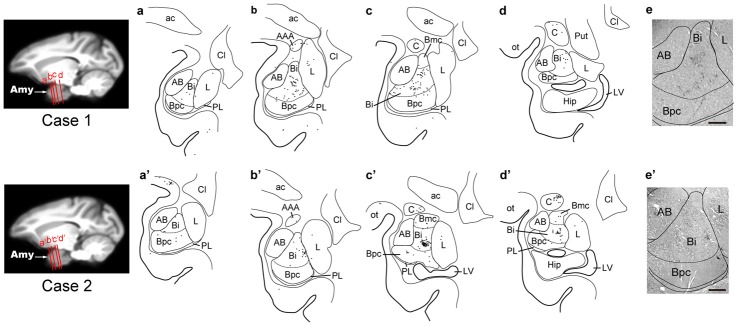
Distribution patterns of the second-order neuron labeling in the amygdala at the 3-day survival period in Case 1 (upper) and Case 2 (lower). In each case, four representative coronal sections are arranged rostrocaudally in **(a–d)** or **(a′–d′)**. The approximate rostrocaudal levels of the sections are indicated in the MRI template brain of the macaque monkey (left). Each dot represents one labeled neuron. Photomicrographs of the sections **(b,b′)** are shown in **(e,e′)**, respectively. AAA, anterior amygdaloid area; Amy, amygdala; Cl, claustrum; Hip, hippocampus; LV, lateral ventricle; ot, optic tract; Put, putamen. Other abbreviations are as in Figures 2, 3.

Three days after the rabies injections into PMv, numbers of neurons were labeled in the amygdala. Of these second-order labeled neurons, almost 80% of the total amygdalar labeling was observed in the basal nucleus (B) (460/569 cells in Case 1 and 542/713 cells in Case 2; Table 2). Within B, a majority of the labeled neurons were located in the intermediate division (Bi) (374/460 cells in Case 1 and 461/542 cells in Case 2; Figure 3H, Table 2). Neuronal labeling in Case 1 was distributed dorsoventrally at the rostral level of Bi (Figure 4, upper), while that in Case 2 occurred somewhat more caudally and formed dense clusters in the central part of the nucleus (Figure 4, lower). A much smaller number of labeled neurons were found in other subdivisions of B, the magnocellular (Bmc; 6.0% of the total amygdalar labeling) and parvocellular (Bpc; 7.0%) divisions (Figure 3H, Table 2). In addition, the accessory basal nucleus (AB) contained only a few labeled neurons (1.5%; Figure 3H, Table 2).

**Table 2:** Distributions of the number (*n*) and ratio (*%*) of labeled neurons in the amygdalar subnuclei after rabies injections into PMv.

Order of transsynaptic transport	Second order			Third order		
Case	Case 1 *n* (*%*)	Case 2 *n* (*%*)	Mean *n* (*%*)	Case 3 *n* (*%*)	Case 4 *n* (*%*)	Mean (*%*)
Basal nucleus							
Bi	374 (65.7)	461 (64.6)	417.5 (65.1)	3271 (52.4)	3678 (51.9)	3474.5 (52.2)
Bmc	37 (6.5)	40 (5.6)	38.5 (6.0)	437 (7.0)	561 (7.9)	499 (7.5)
Bpc	49 (8.6)	41 (5.8)	45 (7.0)	461 (7.4)	602 (8.5)	531 (8.0)
**Subtotal** (Basal nucleus)	460 (80.8)	542 (76.0)	500.5 (78.2)	4169 (66.8)	4841 (68.4)	4505 (67.6)
Accessory basal nucleus (AB)	10 (1.8)	9 (1.3)	9.5 (1.5)	221 (3.5)	268 (3.8)	244.5 (3.7)
Lateral nucleus (L)	27 (4.7)	46 (6.5)	36.5 (5.7)	1078 (17.3)	1059 (15.0)	1068.5 (16.0)
Central nucleus (C)	67 (11.8)	103 (14.4)	85 (13.3)	692 (11.1)	834 (11.8)	768 (11.5)
Other nuclei	5 (0.9)	13 (1.8)	9.0 (1.4)	83 (1.9)	78 (1.1)	80.5 (1.2)
**Total number**	569	713	641	6243	7080	6661.5

Rabies-labeled neurons were further observed in the lateral nucleus (L; 5.7%) and the central nucleus (C; 13.3%; Figure 3H, Table 2). In these subnuclei, neuronal labeling was seen at their caudal levels (Figure 4).

### Labeling of Amygdalar Neurons 4 Days after Rabies Injections into PMv

**Figure 5:**
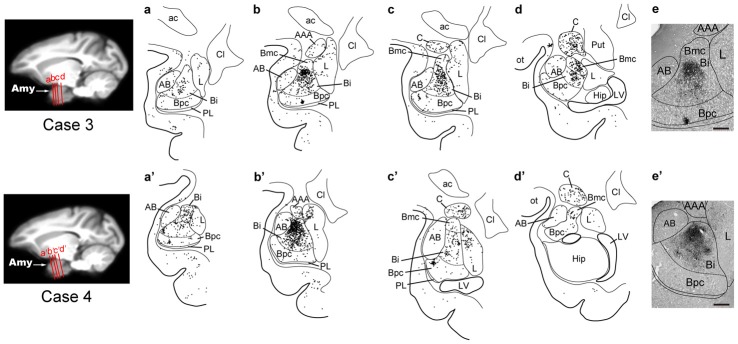
Distribution patterns of the second-order and third-order neuron labeling in the amygdala at the 4-day survival period in Case 3 (upper) and Case 4 (lower). All conventions are as in Figure 4.

By extending the postinjection survival period to 4 days, we explored the possible changes in the distribution pattern of rabies labeling within the amygdala. We found much stronger neuronal labeling appeared in B (67.6% of the total amygdalar labeling; 4169/6243 cells in Case 3 and 4841/7080 cells in Case 4; Table 2). A tremendous number of labeled neurons were located in Bi, consisting of 52.2% of the total amygdalar labeling (3271/6243 cells in Case 3 and 3678/7080 cells in Case 4; Figure 3H right, Table 2). In Case 3, the labeled neurons were distributed dorsoventrally throughout the entire rostrocaudal extent of Bi (Figure 5, upper), while in Case 4, many of the labeled neurons were distributed dorsoventrally in the rostral half of the nucleus (Figure 5, lower).

Compared with the distribution pattern of the second-order neuron labeling in the amygdala, the occurrence of third-order labeled neurons was more prominent in the amygdalar nuclei other than Bi. In Bmc, neuronal labeling was seen primarily in the ventral aspect of a region adjacent to Bi (Figure 5). The number of labeled neurons in Bmc was 499 (7.5%) on average (Figure 3H right, Table 2). In Bpc, the labeled neurons were extensively distributed in the lateral portion of the caudal half of the nucleus (Figure 5). The number of labeled neurons in Bpc was 531 (8.0%) on average (Figure 3H right, Table 2). Much fewer labeled neurons (3.7%) were found in AB (Figure 3H right, Table 2).

Some labeled neurons were also observed in L (16.0% of the total amygdalar labeling) and C (11.5% of the total amygdalar labeling) In L, they were located primarily in the dorsal portion of the caudal half of the nucleus (Figure 3H right, Figure 5, Table 2). In C, the labeled neurons were widely distributed within the nucleus (Figure 3H right, Figure 5, Table 2).

## Discussion

**Figure 6:**
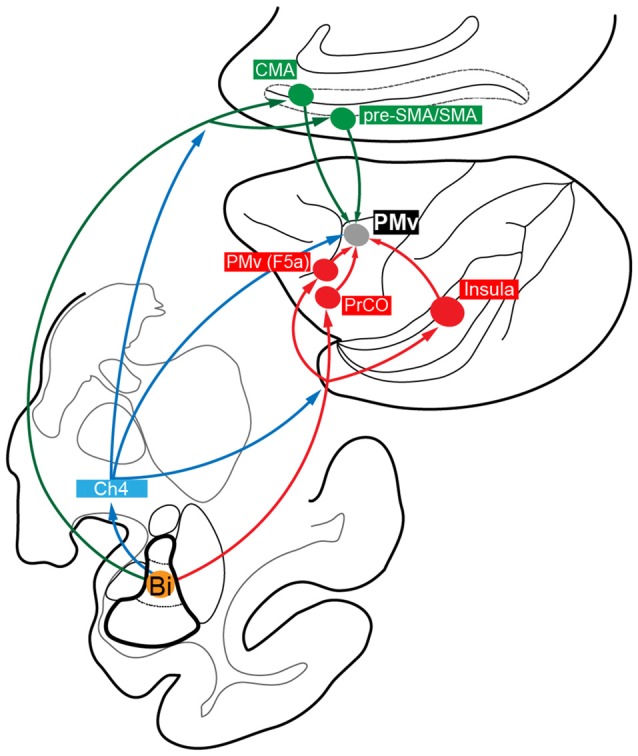
Summary diagram showing the proposed multisynaptic pathways from the amygdala, especially from Bi, to PMv: disynaptic pathways mediated by the medial cortical areas (Bi→CMA/pre-SMA/SMA→PMv; green); disynaptic pathways mediated by the lateral cortical areas (Bi→PrCO/Insula/PMv (F5a)→PMv; red); disynaptic and trisynaptic pathways mediated by Ch4 in the basal forebrain and, then, by the medial and lateral cortical areas (blue).

Many lines of evidence have been accumulated to indicate that the functional interactions between the amygdala and PMv (especially its forelimb region) are indispensable for linking visual-gustatory/somatosensory valence signals to reaching and grasping movements in feeding behavior (Fuster and Uyeda, 1971; Sanghera et al., 1979; Rizzolatti et al., 1981, 1988; Gentilucci et al., 1988; Nishijo et al., 1988a,b). However, no data have so far been available on the direct connectivity from the amygdala to PMv (Jacobson and Trojanowski, 1975; Avendaño et al., 1983; Amaral and Price, 1984). In order to identify possible multisynaptic projections that arise from the amygdala to reach PMv, we employed retrograde transneuronal labeling with rabies virus in macaque monkeys. Our precise histological analysis of the distribution pattern of rabies-labeled neurons in the amygdala has revealed that the basal nucleus (B), particularly its intermediate division (Bi), is the principal origin that connects the amygdala to PMv in a disynaptic fashion. By extending the postinjection survival period from 3 days to 4 days, much greater numbers of labeled neurons were seen in all subnuclei of the amygdala. This might be ascribable to underestimating the second-order neuron labeling with the three-day survival. In addition, the third-order neuron labeling may have appeared at least partly via intraamygdalar connections. As a potential source for third-order neurons innervating Bi, the lateral nucleus of the amygdala (L) can be regarded in view of the fact that unlike other subnuclei, L exhibited a large increase in the ratio of labeled neurons to the total amygdalar labeling. Several cortical and subcortical relays are likely for the architecture of the Bi-derived disynaptic pathways to PMv (Figure 6). However, the possible participation of other multisynaptic pathways may not be ruled out, because rabies labeling technique used in this study cannot necessarily trace all relay sites in a strict fashion though it is a powerful tool for complex neural network analysis.

First, we discuss cortical areas that possibly mediate such disynaptic projections. The forelimb region of PMv, providing a pivotal role in reaching and grasping movements (Rizzolatti et al., 1981, 1988; Gentilucci et al., 1988), receives direct inputs from the medial cortical areas, including CMA, SMA and pre-SMA (Kurata, 1991; Morecraft and Van Hoesen, 1992; Luppino et al., 1993; Dum and Strick, 2005), and from the lateral cortical areas, such as PrCO and Ig/Id (Matelli et al., 1986; Kurata, 1991; Simonyan and Jürgens, 2002, 2005). Previous anatomical studies have demonstrated that the PMv interactions with the medial and lateral cortical areas may crucially be involved in motor actions by the hand and mouth (Rozzi et al., 2006; Borra et al., 2008, 2011; Gerbella et al., 2011, 2013, 2016a). It has been well documented that these cortical areas have projections directly from Bi as well as from the magnocellular division of B (Bmc; Pandya et al., 1973; Jacobson and Trojanowski, 1975; Mufson et al., 1981; Porrino et al., 1981; Avendaño et al., 1983; Amaral and Price, 1984; Vogt and Pandya, 1987; Friedman et al., 2002; Morecraft et al., 2007; Yukie et al., 2010; Gerbella et al., 2016b). The medial cortical areas receive additional input from the accessory basal nucleus (AB), while the lateral cortical areas receive those from the parvocellular division of B (Bpc), the central nucleus of the amygdala (C) and the dorsal portion of L (Mufson et al., 1981; Porrino et al., 1981; Amaral and Price, 1984; Friedman et al., 1986; Morecraft et al., 2007). Altogether, it is most likely that the amygdala, especially Bi, sends disynaptic projections to PMv by way of the medial and lateral cortical areas (Figure 6).

Second, we discuss subcortical structures that mediate the disynaptic projections from Bi to PMv. It has been reported that B, including Bi, projects directly to the cholinergic cell group 4 (Ch4) in the basal forebrain (Russchen et al., 1985a,b; Aggleton et al., 1987; Fudge and Tucker, 2009). Two subgroups of Ch4 neurons have been shown to constitute the major sources for cholinergic innervation of widespread cortical areas. The antero-lateral/medial territory (Ch4al/am) projects to the medial cortical areas as well as to the premotor areas including PMv, while the intermedio-dorsal/ventral territory (Ch4id/iv) projects to the lateral cortical areas (Kievit and Kuypers, 1975; Jones et al., 1976; Mesulam et al., 1983). In fact, we found that labeled neurons (probably both the first-order and the second-order labeling) were distributed predominantly in Ch4al/am and, to a lesser extent, in Ch4id/iv 3 days after the rabies injections into PMv (see Figure 2B). Conceivably, these Ch4 neurons may at least partly contribute to possible trisynaptic pathways linking Bi to PMv through the medial and lateral cortical areas (Figure 6).

Thus, it has been revealed in the present study that the Bi-derived multisynaptic pathways to PMv consist of three distinct routes via the medial and lateral cortical areas and, further, via Ch4 in the basal forebrain. With respect to the medial cortical areas, CMA, especially its rostral part, plays an important role in the monitoring of movements as well as in the selection of forthcoming behaviors based on the reward value (Niki and Watanabe, 1979; Shima et al., 1991; Devinsky et al., 1995; Shima and Tanji, 1998b; Matsumoto et al., 2003; Walton et al., 2004; Hoshi et al., 2005; Buckley et al., 2009), while pre-SMA is closely related to the planning, preparation and execution of movements based on the visual information (Halsband et al., 1994; Tanji and Shima, 1994; Matsuzaka and Tanji, 1996; Hoshi and Tanji, 2004; Nachev et al., 2008). Concerning the lateral cortical areas, it has repeatedly been reported that the posterior insular cortex (i.e., Ig/Id) guides hand manipulation and ingesting behaviors according to the somatosensory and visceral information (Mesulam and Mufson, 1982; Mufson and Mesulam, 1982; Schneider et al., 1993; Augustine, 1996; Ishida et al., 2013), and that both PrCO and Ig/Id receive projection fibers directly from the primary gustatory cortex (Pritchard et al., 1986; Yaxley et al., 1990) and are involved not only in mastication, but also in sensory processing of the oral cavity (Ogawa et al., 1989). For feeding behavior, it is crucial to evaluate an object to reach for and bring to the mouth. One intriguing idea is that CMA/pre-SMA neurons might use a valence signal about the object derived from Bi in selecting and determining a motor action based on the object value, whereas PrCO and Ig/Id might integrate the valence signal with the gustatory-somatosensory information to prepare grasping and eating actions. In addition, SMA and pre-SMA could use the valence signal to control the temporal organization of multiple actions in feeding behavior (Shima et al., 1996; Shima and Tanji, 1998a; Tanji, 2001). Moreover, the forelimb region of PMv possesses a dense intrinsic connection with the more rostral and ventral sector of PMv (corresponding to F5a; Belmalih et al., 2009; Gerbella et al., 2011; see also Figures 1Eb,Fb′), although its functional role remains unknown. Since seminal studies reported that F5a received projection fibers directly from B and AB of the amygdala (Ghashghaei et al., 2007; Yukie et al., 2010), this PMv area may serve as an interface between the amygdala and the forelimb region of PMv in feeding behavior (Gerbella et al., 2011; Borra et al., 2017). Furthermore, it has been well documented that a region of PMv contains so-called “mirror neurons” (Rizzolatti et al., 1996; Ferrari et al., 2003), and that the amygdala participates in social cognition (Rizzolatti et al., 2014). This implies that the amygdalar projections to PMv may contribute to social behaviors, for example, social communication based on subjective value of observed action (Caggiano et al., 2012).

Previous anatomical works have shown that L but also Bmc/Bi of the amygdala receives massive input from the sulcal region of area TE (Herzog and Van Hoesen, 1976; Iwai and Yukie, 1987; Webster et al., 1991; Ghashghaei and Barbas, 2002; Amaral et al., 2003), known to represent the shape and surface texture of a three-dimensional object (Janssen et al., 2000; Sereno et al., 2002), and that C of the amygdala receives gustatory input from the insular cortex (Bermúdez-Rattoni et al., 2004; Cai et al., 2014). These amygdalar subnuclei intensively communicate with Bi, which has been implicated in visuo-gustatory integration for feeding behavior (Sanghera et al., 1979; Nishijo et al., 1988a,b; Fudge et al., 2002). In conjunction with the visuo-gustatory valence signal, the attentional and motivational aspects have been reported to influence feeding behavior (Mogenson et al., 1980). Neurons in Ch4 are selectively responsive to novel alimentary stimuli (Wilson and Rolls, 1990) and change their firing rate in response to the sight or taste of food according to the state of hunger (Burton et al., 1976). Furthermore, the PMv participates in spatial attention for visual guidance of motor behaviors (Kubota and Hamada, 1978; Rizzolatti et al., 1983, 1988; Godschalk et al., 1985; Boussaoud and Wise, 1993; Schieber, 2000) and represents a context-dependent motivational signal for action (Roesch and Olson, 2003, 2004). These observations suggest that PMv may receive attentional and motivational signals from Ch4 in the basal forebrain. Another origin of the attentional and motivational signals of action could be the basal ganglia. In our prior study (Ishida et al., 2016), we have elucidated that the forelimb region of PMv receives inputs from the motor and limbic territories of the globus pallidus (GP) and, then, from the ventral striatum including the nucleus accumbens, each of which is a target of the Ch4-derived projection (Haber et al., 1990; Spooren et al., 1996). Taken together, PMv is considered to receive diverse attentional and motivational signals from Ch4 and the basal ganglia that may help PMv to control multiple forelimb movements for feeding behavior.

## Author Contributions

HI analyzed data and wrote the present manuscript. KI performed injection experiments. KI and MT critically read and edited the manuscript.

## Conflict of Interest Statement

The authors declare that the research was conducted in the absence of any commercial or financial relationships that could be construed as a potential conflict of interest.
